# The Calabrian Arc: three-dimensional modelling of the subduction interface

**DOI:** 10.1038/s41598-017-09074-8

**Published:** 2017-08-21

**Authors:** Francesco E. Maesano, Mara M. Tiberti, Roberto Basili

**Affiliations:** Istituto Nazionale di Geofisica e Vulcanologia, Via di Vigna Murata 605, 00143 Rome, Italy

## Abstract

The Calabrian Arc is a one-of-a-kind subduction zone, featuring one of the shortest slab segments (<150 km), one of the thickest accretionary wedges, and one of the oldest oceanic crust in the world. Despite a convergence rate of up to 5 mm/y and well-known intraslab seismicity below 40 km, its shallow interface shows little signs of seismic activity. Nonetheless, it has been attributed as generating historical large earthquakes and tsunamis. To gain insights into this subduction zone, we first made a geological reconstruction of the shallower slab interface (<20 km) and its overlying accretionary wedge by interpreting a grid of 54 seismic reflection lines (8,658 km) with 438 intersections within an area of 10^5 ^km^2^. Then, we constrained a deeper portion of the slab surface (40–350 km) using the seismicity distribution. Finally, we interpolated the two parts to obtain a seamless 3D surface highlighting geometric details of the subduction interface, its lateral terminations and down-dip curvature, and a slab tear at 70–100 km depth. Our 3D slab model of the Calabrian Arc will contribute to understanding of the geodynamics of a cornerstone in the Mediterranean tectonic puzzle and estimates of seismic and tsunami hazards in the region.

## Introduction

Megathrust earthquakes occur on the seismic interface of subduction zones and many of them generate significant tsunamis^1–4^. The maximum potential earthquake magnitude in subduction zones depends on several factors^5^, including the geometry of the seismic interface. For example, anticorrelation is found between subduction interface curvature and maximum earthquake magnitude^2^. The along-dip shape also controls the uplift/subsidence pattern in individual earthquakes^4^ and over multiple earthquake cycles^6, 7^. In the presence of low-velocity sedimentary wedges, the shallowest portion of subduction interfaces near the toe of the wedge can also generate tsunami earthquakes^1, 8^. The geometry of the slab interface is also used for making considerations on geodynamic processes based on its relations to buoyancy, length, and edge instability^9^.

**Figure 1:**
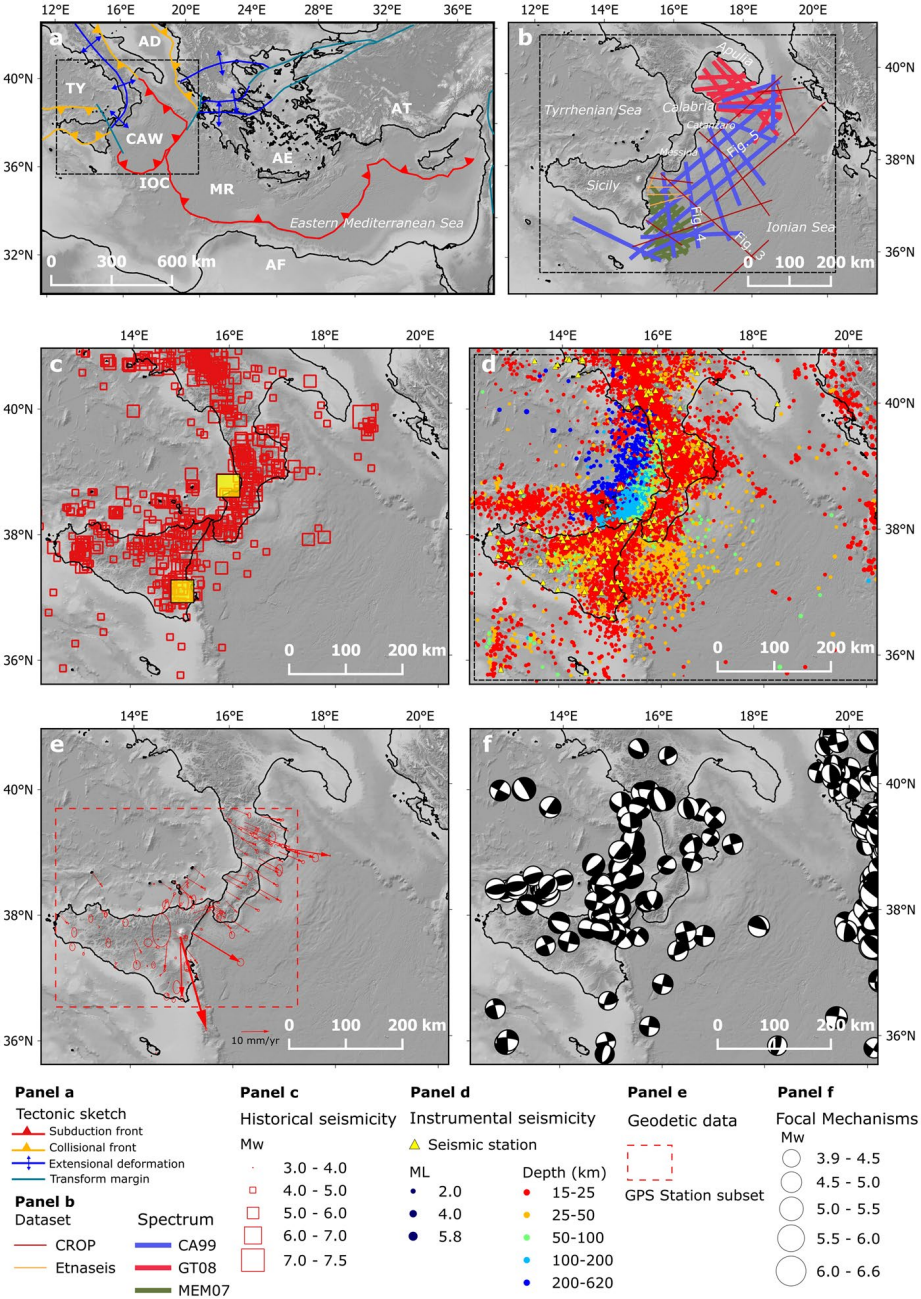
Map of the study area. Panel a – Tectonic sketch of the Eastern Mediterranean region; AF: African plate, AE: Aegean plate, AT: Anatolian plate, EU: Eurasian plate, AD: Adria microplate, CAW: Calabrian accretionary wedge; IOC: Ionian oceanic crust, MR: Mediterranean ridge, TY: Tyrrhenian Sea. The dashed rectangle shows location of Panel b. Panel b - Dataset used in this work. Seismic profiles from: CROP Project (http://www.crop.cnr.it/front-page_EN), Spectrum (http://www.spectrumgeo.com/ provided under confidentiality agreement CA60), Etnaseis survey^42^. Panel c – Historical seismicity from CPTI15^69^. Panel d – Instrumental seismicity from the Italian Seismological Instrumental and Parametric Database^68^, earthquake plotted are recorded in the time period 2005–2016. Panel e – Velocity field from continuous GPS station in the 1998–2009 time span for Sicily and Calabria plotted with a fixed Africa ref. 70. Velocity ellipses represent 1-sigma confidence errors. Panel f – Regional Centroid Moment Tensor solution^71^. Topo-bathymetric relief (Panels a-f) is obtained from SRTM30_PLUS^72^. Coastlines are from the European Environmental Agency (http://www.eea.europa.eu/)

The subduction zone of the Calabrian Arc is a turning point along the roughly E–W Eurasia-Africa plate boundary in the Central Mediterranean (Fig. 1). Here the subduction of oceanic crust began about 80 Ma ago^10^ and currently continues along a rather small sector (~150 km) of the arc between the Isthmus of Catanzaro to the north, and the Strait of Messina to the south (Fig. 1). The evolution of this sector of the arc is controlled by the slab roll-back that started in the late Miocene (8–10 Ma)^11, 12^, due to the sinking of the Ionian Mesozoic oceanic crust, proposed to be the oldest oceanic crust worldwide (280 Ma)^13, 14^. The effect of the NNW-SSE plate convergence upon subduction gradually decreased with the progressive rotation and south-eastward migration of the Calabrian Arc due to continental collision in Sicily^15^. At present, the subduction process controls both the south-eastward migration and the seismotectonics of the upper plate^16–18^.

Despite a convergence rate of up to 5 mm/y^19, 20^, and well-known intraslab seismicity below 40 km, the shallow seismicity recorded in the Ionian offshore region is very limited in frequency and magnitude. Large historical earthquakes, however, have sometimes been associated with the shallow portion of the plate interface or with the slab, such as the south-eastern Sicily, Mw 7.3, earthquake of 1693^21^ and the Central Calabria, Mw 7.0, earthquake of 1905^22^ (Fig. 1). Despite its relatively small size, the Calabrian Arc is considered as a potential source for large earthquakes and, consequently, for seismic and tsunami hazards^5, 23–27^ in a highly populated region.

Seismic profile interpretation has been widely used to reconstruct the shallow part of the Calabrian subduction interface and the main features of its accretionary wedge^17, 28–36^, its interaction with the Apulian platform^37–40^, and its lateral terminations^36, 41–47^. All available reconstructions of the shallow portion of the subduction interface and of the accretionary wedge are based on the interpretation of individual 2D seismic profiles. Among them, the most complete view of the accretionary wedge focuses on the interpretation of its main crustal structures by means of a series of profiles and a 3D map of the Miocene-Quaternary syn-tectonic basins^32^. The ongoing activity of the lateral slab tear fault off eastern Sicily with dextral displacement, folding and thrusting processes caused by active subduction is demonstrated through a detailed morphology of the Calabrian accretionary wedge combining multi-beam bathymetry and high-resolution seismic profiles^48^.

In the deeper region (40–600 km), the slab has a steeply-dipping geometry well depicted by seismic tomography^49, 50^ and the distribution of seismicity^51^. The south-western lateral edge of the slab is marked by an abrupt ending of the deep seismicity^52^ which ideally projects toward an important NW-SE trending structure classified as a Subduction Transform Edge Propagator (STEP, *sensu* Govers and Wortel^53^)^33, 46–48^. Toward the north-east, the seismicity also ends abruptly beneath the Isthmus of Catanzaro^54^ and indicates the presence of a slab window at depths between 100 and 200 km^50, 51^.

In order to capture the three-dimensional complexity of the Calabrian Arc we propose a novel 3D reconstruction of the Calabrian Subduction Interface (CSI). The shallower part (4–18 km) of the subduction interface is based on a comprehensive interpretation of a dense network of seismic reflection lines (Fig. 1b and Table 1), most of which were unavailable in previous studies. The whole network of interpreted seismic lines is simultaneously depth-converted applying the Vel-IO 3D technique^55^ using an instantaneous-velocity model of the Calabrian Accretionary Wedge (CAW). The geometry of the deeper region, up to about 300 km depth, is based on the distribution of seismicity and is compared with tomographic images by Neri, *et al*.^50^ for consistency. The shallow and deep reconstructions are then merged together through interpolation to obtain a seamless unified model of the slab interface. Our 3D reconstructions complement or update the collection of three-dimensional geometries of subduction zones worldwide including the Slab 1.0^56^, DISS^57^, and EDSF^58^ databases and provide a ready-to-use slab model for better constraining future seismic and tsunami hazard analyses^25, 27, 59, 60^ and geodynamic models in the central Mediterranean^10, 12, 16, 20^.

**Table 1:** Characteristics of the seismic reflection dataset.

Survey	Number of lines	Total length (km)	Record length (s)	Acquisition parameters						
				Source type	Source power (c.i.)	Source depth (m)	Streamer depth (m)	Streamer length (m)	Shotpoint interval (m)	Group interval (m)
CA99	16	3391.4	6–12	Airgun Array	3410	6	8	6000	25	12.5
MEM07	15	1530.5	12	Airgun Array	2200	6	8	7200	37.5	12.5
GT08	12	1712.6	8	Airgun Array	5000	6	8	8100	25	12.5
CROP	6	1625.6	17–20	High Pressure Airgun	140 bar	6	12	4500	62.5	25
Etnaseis	5	398.2	11–17	Airgun array	840 3810	23	20^1^ 21^2^	4475^1^ 2375^2^	42.5^3^ 45^4^ 50^2^	25

Notes The acquisition source power is conventionally expressed in cubic inches (c.i.) except for the CROP survey (bar).

Etnaseis seismic line numbers as in Nicolich, *et al*.^42^: ^1^E1, E2, E6; ^2^E3, E5; ^3^E6; ^4^E1, E2.

## Results

### The subduction interface: shallow region (4–18 km depth)

#### Seismic reflection dataset

We built a seismic reflection dataset by merging three subsets of data (Fig. 1b). The first, and more extensive, subset consists of three surveys provided by Spectrum Geo Ltd (http://www.spectrumgeo.com/) to INGV under a collaboration regulated by a non-exclusive Confidentiality Agreement, namely: CA99 which covers the entire study area, MEM07 which adds details in the south-west (offshore eastern Sicily), and GT08 which adds details in the north-east (Taranto Gulf). The second subset of data contains seismic profiles straddling the entire study area, acquired by the CROP Project during the 1990’s for the investigation of the deep crust in Italy (http://www.crop.cnr.it/). The third subset of data comes from the Etnaseis survey, covering a limited area offshore the Etna Volcano^42^. The main characteristics of the whole dataset are summarized in Table 1. Altogether, the dataset consists of 54 seismic reflection lines for a total of 8,658.3 km of surveys within an area of 99,043.2 km^2^. The grid of seismic lines includes 438 intersections which are critical points to check the consistency of the data, especially when they come from different surveys.

#### Seismo-stratigraphy dataset

**Figure 2:**
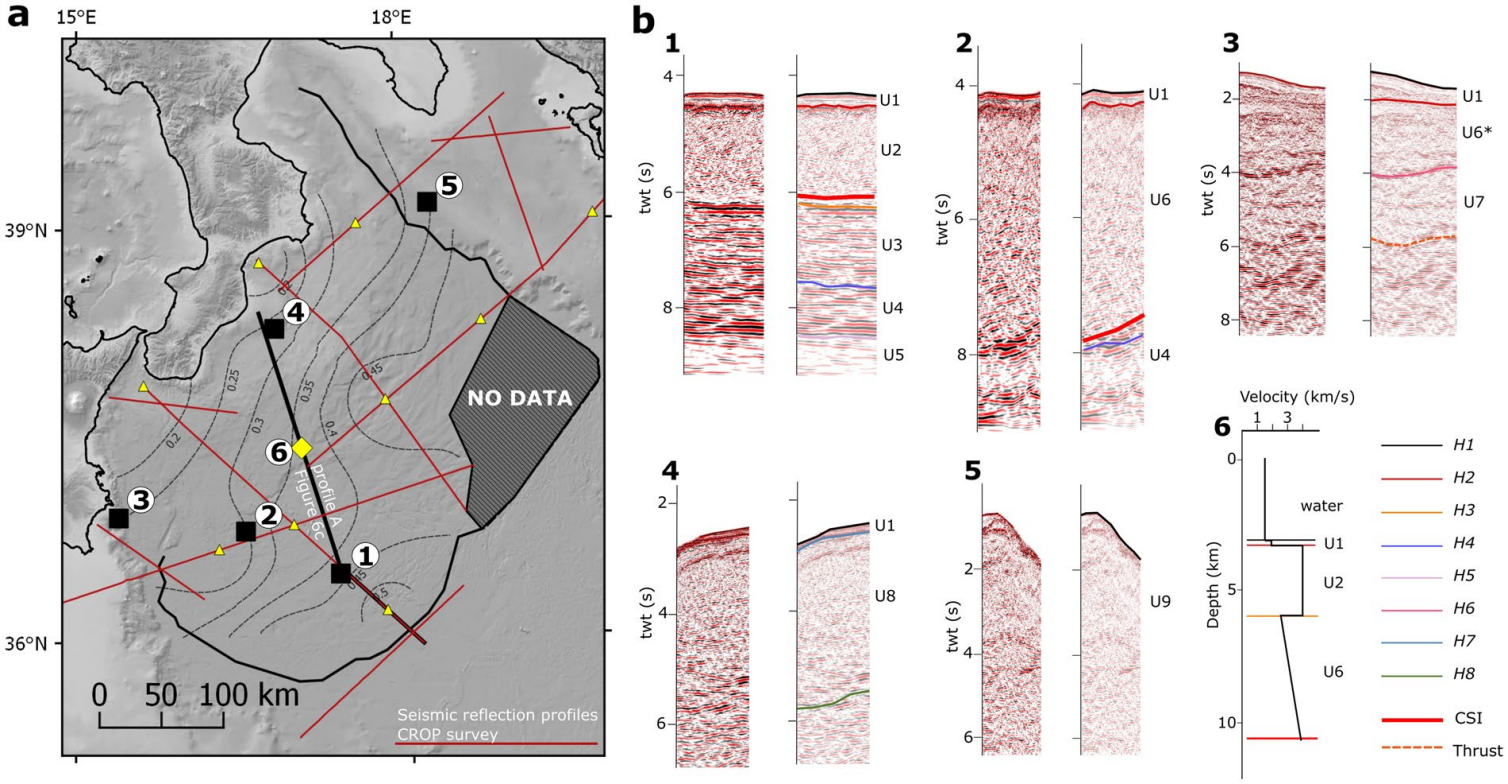
Seismo-stratigraphic scheme and velocity model. Panel a – Map of the sample seismo-stratigraphic logs (Panel b, #1-5) and the velocity gradient (k, labelled dashed contour lines) within U6 (Panel b, #6). The location of points used for analysis of the stacking velocities is shown by yellow triangles; the location of the sample velocity model is indicated by the yellow diamond. Panel b - Sample seismo-stratigraphic logs (#1-5) and sample velocity model (#6). Description of units (U1-9) and bounding horizons (H1-7) are reported in Tables 2 and 3, respectively. Notice that U6* in this sample stratigraphy represents the thrust top basin sediments deposited over U7; U6 and U7 are both included in the lowermost layer (L4) of the velocity model (Table 4). Topo-bathymetric relief is obtained from SRTM30_PLUS^72^. Coastlines are from the European Environmental Agency (http://www.eea.europa.eu/). Raw seismic data provided by Spectrum Geo (http://www.spectrumgeo.com/).

**Figure 3:**
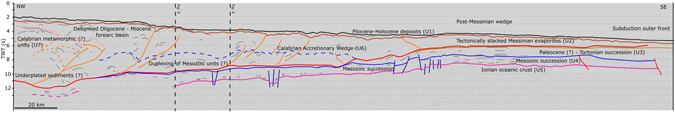
Structural interpretation of the CROP M2B seismic profile line drawing. Horizon colour codes as in Fig. 2. Structural elements: Orange lines: contractional structures within the accretionary wedge. Red lines: subduction interface. Dark blue lines: extensional or transtensional faults. Dashed vertical lines indicate the intersection with profile #7 (Fig. 4 and Supplementary Figure S2) and profile #14 (Fig. 5 and Supplementary Figure S3).

We identified nine seismo-stratigraphic units (U1–9), with correlative geologic units derived from previous works by several authors, and eight horizons (H1–8). Figure 2, Tables 2, and 3 show the main characteristics of the identified seismic facies and their bounding horizons. U1-5 and H1-5 were first identified in the undeformed abyssal plain of the Ionian basin (CROP line M2B, Fig. 3) and then mapped throughout the study area. The remaining units and horizons are represented only locally. In particular, U6 is the combination of U3, U4, U7, and possibly various other units of the sequence, deformed in the tectonic melange that forms the accretionary wedge. The velocity model derived from this dataset (Table 4, Fig. 2) is composed of four layers; notice that only U1, U2, and U6 are used for the depth conversion to map the subduction interface.

**Table 2:** Seismo-stratigraphic units.

ID	Seismo stratigraphic facies	Age	Description	References
U1	Well layered HF-LA reflectors with presence of numerous unconformities.	Pliocene - Holocene	Unconsolidated siliciclastic turbiditic and distal deposits often associated with chaotic bodies at the base of the succession.	32, 33, 36
U2	HA and quite continuous reflectors at the top, overlying a highly homogeneous reflection-free zone bounded at the base by a planar HA reflector.	Messinian	Salt-bearing complex related to the tectonic stacking of Messinian evaporites.	33, 34, 75
U3	Alternating HA-LF, subparallel reflectors, bounded at the top by a continuous HA reflector; this unit can be differentiated from the accretionary wedge only in the external regions.	Paleocene (?) - Tortonian	Siliciclastic deposits (analogue of outcropping units in Sicily and Southern Apennines) marked by on-lap on U4.	32, 33, 76
U4	LA-LF subparallel reflectors characterized at the base by high-amplitude reflectors 0.3 s TWT thick.	Triassic - Cretaceous	Pelagic carbonate sediments (analogue of outcropping units in Sicily and Southern Apennines), covering the Ionian oceanic crust.	34, 75
U5	Noisy seismic facies located under a HA-LF seismic reflections at 8.5 s TWT in the external area of the accretionary wedge.	Permian (?) - Triassic	Ionian oceanic crust.	13, 29, 77
U6	Chaotic and highly deformed LA seismic facies with some LF-HA discontinuous reflectors.	Mesozoic - Pliocene	Calabrian Accretionary Wedge made up by the tectonic stacking of U3, U4, and U7.	32–34
U7	Transparent seismic facies bounded at the top and the base by HA reflectors.	n.a.	Calabrian metamorphic (?) unit.	32–34
U8	Mainly transparent seismic facies bounded by HA reflectors related to erosional unconformity at the top and by LF-HA reflectors at the base.	Mesozoic - Messinian	Apulia platform carbonates.	31–39
U9	Layered HF seismic facies in the upper part; transparent seismic facies in the lower part.	Mesozoic - Miocene	Hyblean platform carbonates.	41–43

Notes: HA = high amplitude; LA = low amplitude; HF = high frequency; LF = low frequency.

**Table 3:** Horizons.

ID	Description	References
H1	Seafloor reflector.	n.a.
H2	Regional angular unconformity at the top of U2. Where U2 is absent, it represents the top of U6.	32, 33
H3	HA reflector representing the unconformity and correlative conformity at the top of U3.	33, 34, 76
H4	Planar and continuous reflector representing the top of U4.	32, 75
H5	HA-LF reflectors identified in most of the seismic lines and representing the top of U5.	34
H6	HA-LF reflector, generally dipping seaward, interpreted as the top of U7.	32, 33
H7	Intra-Messinian unconformity recognized in all the seismic profiles crossing U8.	31
H8	HA-LF reflectors at the base of U8.	30

Notes: HA = high amplitude; LF = low frequency.

**Table 4:** Velocity model.

Layer	Layer name	Unit	V (m/s)	K (1/s)
L1	Water	n.a.	1500	0
L2	Plio-Pleistocene unit	U1	1900	0
L3	Messinian wedge	U2	4000	0
L4	Pre-Messinian wedge and units	U6	2500	0.15–0.50

#### Time-domain model

**Figure 4:**
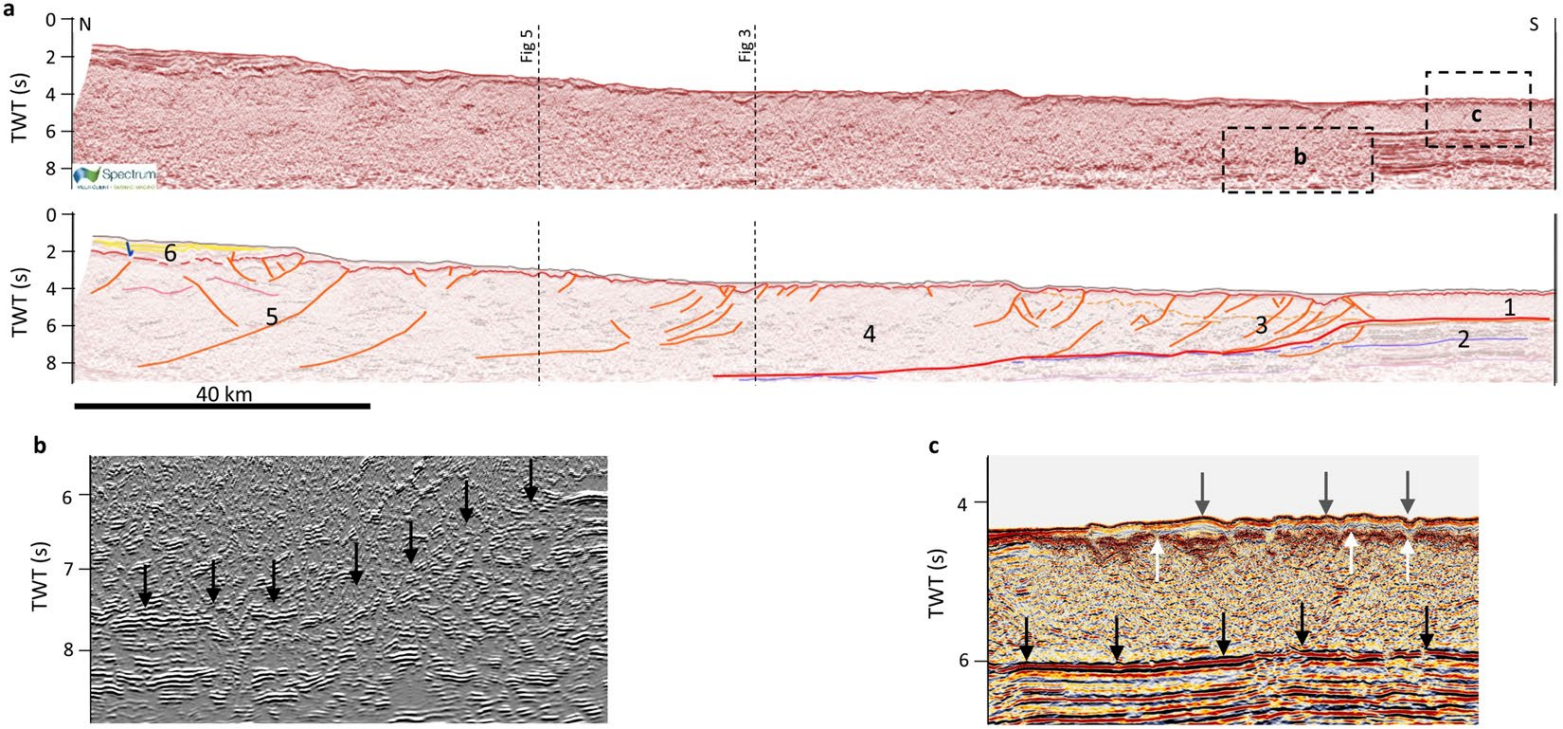
NW-SE seismic profile interpretation. Panel a - Seismic profile (profile #7 in Supplementary Figure S2). Horizon colour codes as in Fig. 2. Description of units (U1-9) and bounding horizons (H1-7) are reported in Tables 2 and 3, respectively. Yellow lines: unconformities within the Plio-Holocene basins (U1). Structural elements: Orange line: contractional structures within the accretionary wedge. Red line: subduction interface. Dark blue lines: extensional or transtensional faults. Key elements for the interpretation: 1 – Post-Messinian wedge; 2 - Relatively undisturbed sedimentary filling (U3 and U4) of the Ionian basin; 3 - Frontal splays (presumably involving U2); 4 - Accretionary wedge (U6); 5 - Inner thrust involving U7; 6 - Plio-Holocene forearc basin. Dashed vertical lines indicate the intersection with profile #14 (Fig. 5 and Supplementary Figure S3) and profile CROP M2B (Fig. 3). Panel b – Detail of the seismic line in a display using the energy attribute showing the position of the subduction interface (black arrows). Panel c – Detail of the seismic line showing the position of the subduction interface (black arrows), the deformed unconformity H2 (white arrows), and the deformed seafloor H1 (grey arrows). Raw seismic data provided by Spectrum Geo (http://www.spectrumgeo.com/).

**Figure 5:**
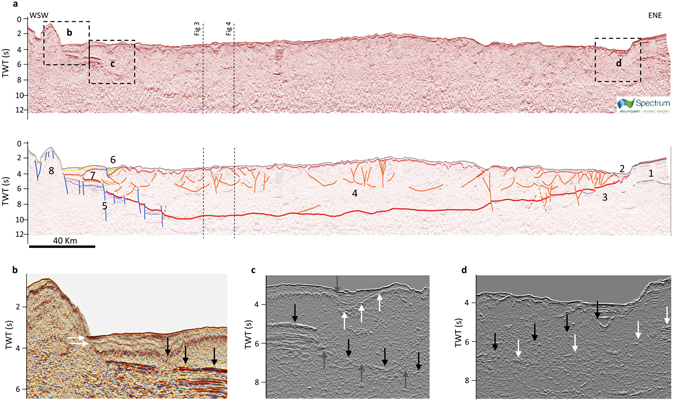
SW-NE seismic profile interpretation. Panel a - Seismic profile (profile #14 in Supplementary Figure S3). Horizon colour codes as in Fig. 2. Description of units (U1-9) and bounding horizons (H1-7) are reported in Table 2 and Table 3, respectively. Yellow lines: unconformities within the Plio-Holocene basins (U1). Structural elements: Orange lines: contractional structures within the accretionary wedge. Red lines: subduction interface. Dark blue lines: extensional or transtensional faults. Key elements for the interpretation: 1 – Apulia Platform (U8); 2 – Plio-Holocene foredeep; 3 – U8 at the footwall of the outer accretionary wedge (U6); 4 – Accretionary wedge (U6); 5 – Subduction Transform Edge Propagator (STEP) fault system; 6 – Syn-tectonic Plio-Holocene extensional basin related to the STEP fault; 7 – Post-Messinian wedge; 8 – Hyblean Platform (U9). Dashed vertical lines indicate the intersection with profile #7 (Fig. 4 and Supplementary Figure S2) and profile CROP M2B (Fig. 3). Panel b - Detail of the seismic line showing the position of the subduction interface (black arrows) and show the stratigraphic onlap of the undeformed U1 (to the left) onto the Malta Escarpment (to the right; Fig. 6). Panel c - Detail of the seismic line in a display using the energy attribute showing the position of the subduction interface (black arrows) and its local displacement by sub-vertical faults (grey arrows pointing up), and the unconformity H2 (white arrows) deformed by a shallow normal fault (grey arrow pointing down). Panel d - Detail of the seismic line in a display using the energy attribute showing the position of the subduction interface (black arrows), and the position of a high-energy reflector within U8 followed under the subduction interface (white arrows). Raw seismic data provided by Spectrum Geo (http://www.spectrumgeo.com/).

The CSI was identified with a sharp change in amplitude and dip of the reflectors marking the boundary between the Ionian Oceanic Crust (IOC) and the overlying CAW (Figs 4a and 5a). The CSI is often associated with a decrease of the signal amplitude and with curled reflectors that can be interpreted as having been dragged along the interface itself. The CAW is generally characterized by discontinuous and curled reflectors and by small signal-to-noise ratio. Within the CAW, the seismo-stratigraphic units illustrated in Table 2 cannot be distinguished from one to another except for U1. Conversely, the IOC is characterized by sub-horizontal or slightly dipping and more continuous low-frequency reflectors.

The reflectors corresponding to the top of the IOC (H5 in Table 2), also constrained using the low-pass filter and the energy attribute (Supplementary Figure S1), corroborate the continuity of the lower plate (Figs 3b, 5c, and d) that, unlike reflectors within the CAW (U6), is characterized by a high content of low-frequency, high-amplitude, and high-energy signals.

Based on the seismic characteristics outlined above, we could recognize the CSI in all the seismic lines (Supplementary Figures S2 and S3). With reference to the profile reported in Fig. 3, the following main structural features characterize the CSI from SE to NW. 1) A detachment running at the base of U2 in the south-eastern sector. The propagation of the deformation along the CSI in this sector determines the thickening of the evaporites and the folding of the overlying U1. The average thickness decreases southeastwardly. 2) A step-like ramp in the central sector cutting through U3, U4, U5, and U6 and tapering northwestwardly just above H4 (Fig. 4b and seismic profiles #6 and #7 in Supplementary Figure S2). 3) Another detachment running just above H4 in the north-western sector. Notice that above this detachment, deformed U4 slices may constitute a duplex structure. This detachment can be followed in the orthogonal direction in Fig. 5a and Supplementary Figure S3, which also shows its north-east and south-west lateral terminations. 4) Another ramp, though not very well defined and not imaged in all the available seismic profiles, cutting through the lower plate and where U4 could be partly or completely detached from the IOC and underplated. This sector also presents a systematic velocity pull-up effect of the CSI, probably related to the presence of the high-velocity U7 (see also Supplementary Figure S2).

The time-domain model is made available as an XYZ spreadsheet (Supplementary file CSI_TDM.XLS).

#### Depth-domain model

**Figure 6:**
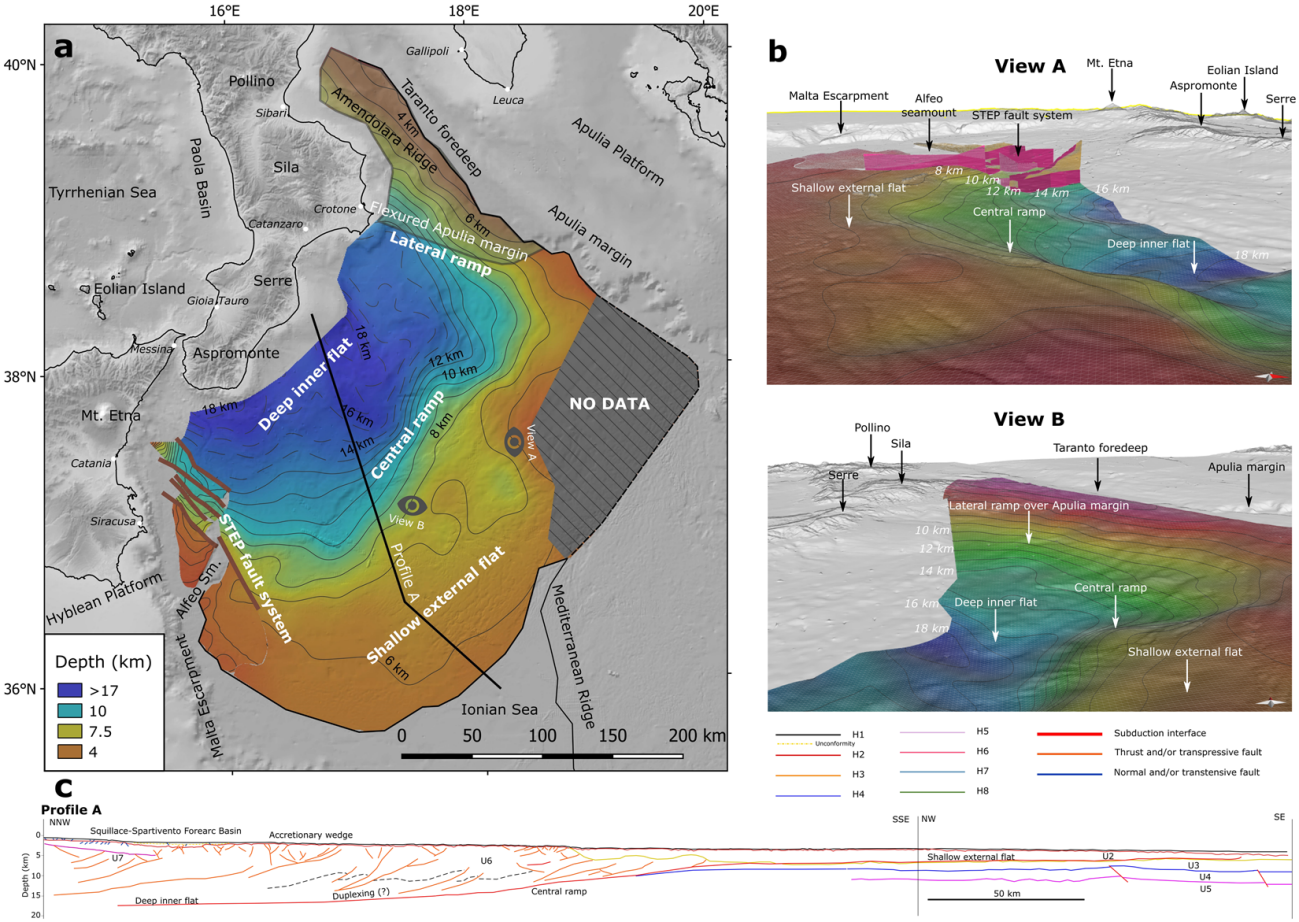
Depth-domain model of the shallow part of the subduction interface. Panel a – Gradient-colour map and depth contours (dashed where affected by larger uncertainty) of the shallow subduction interface (smoothing distance = 25 km), brown lines along the STEP fault system represents recent/active transtensional fault segments; Panel b – Perspective views of the subduction interface, viewpoints are indicated in Panel a. Panel c - Cross section (trace in Panel a) of the subduction interface and of the internal structure of the CAW from the interpretation and depth conversion of the seismic reflection profiles. Topo-bathymetric relief (Panels a and b) and profiles (Panel c) are obtained from SRTM30_PLUS^72^. Coastlines are from the European Environmental Agency (http://www.eea.europa.eu/).

Based on a 3D depth conversion of the CSI, using the interpretation of all the seismic lines illustrated in the previous paragraph and the velocity model shown in Table 4 and Fig. 2, we identified five main key features. With reference to the reconstruction shown in Fig. 6, these features are: 1) shallow external flat, 2) deep inner flat, 3) central ramp, 4) lateral ramp, and 5) STEP fault system.

The shallow external flat is a wide area between 5 and 8 km depth corresponding to the propagation of the CSI at the base of the tectonically stacked Messinian evaporites (U2) and extends all around the south-eastern side of the CSI (Fig. 6c).

The deep inner flat is a low-angle feature (ca. 4°) located at depths between 14 and 19 km in the central part of the mapped interface. Unlike the shallow external flat, this feature shows a higher geometrical variability. In particular, two main NW-SE elongated lows alternate with two highs, transversely altering the general NW dipping attitude of the CSI.

The central ramp is a steep portion of the CSI between 8 km and 14 km depth that connects the two flats just described. The slope of the ramp shows a significant change in the central part of the CSI. The northern part of the ramp is steeper (up to 18°) and curved (with aspect varying in a clockwise sense from SW-directed to NW-directed). The southern part of the ramp is gentler (ca. 7°) and straighter. The connection between the two sectors of the ramp forms a knob in front of the lowermost part of the deep inner flat.

The lateral ramp is a steep portion of the CSI elongated in a NW-SE direction between 4 km and 10 km depth, and juxtaposing the allochthonous units of the CAW over the flexured Apulia margin (Fig. 5d and profiles #1 and #2 in Supplementary Figure S2 and profiles #14 and #15 in Supplementary Figure S3). The Apulian units (U8) could be followed a long distance under the CAW up to the Crotone offshore (profile #3 in Supplementary Figure S2). In the north-western sector of the lateral ramp the CSI is deformed by transpressive structures (Amendolara Ridge) related to the collisional process between the upper plate and the Apulia Platform (profiles #1 and #2 in Supplementary Figure S2). At the tip of the lateral ramp, the outer front of the CSI deforms the Taranto foredeep, filled in by up to 1000-meter Plio-Holocene deposits onlapping the Messinian unconformity of the Apulia Platform (Fig. 5d).

The STEP fault system consists of NW-SE high-angle faults, NE-side down, that interrupt the lateral continuity of the CSI in the south-western side (Fig. 5c). The STEP fault system cuts the entire IOC (U5) at least down to the Moho, with decreasing displacement and variable structural architectures along strike from NW to SE (compare profiles #11, #13, #14, and #15 in Supplementary Figure S3). In particular, it shows a complex fault array to the north of the Alfeo Seamount that also affects the internal structure of the CAW, and a single major fault from the Alfeo Seamount toward the south. The shallow external flat is only partially affected by the STEP and encircles its southernmost tip.

The depth-domain model is made available as an XYZ spreadsheet (Supplementary file CSI_DDM.XLS).

### The subduction interface: deep region (40–300 km depth)

**Figure 7:**
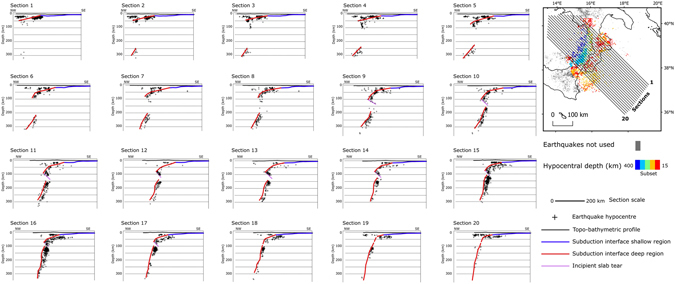
Seismicity distribution and reconstruction of the subduction deep region. Cross sections used for the interpretation of the deep slab. Section traces shown in map (upper-right corner). Earthquakes used for this interpretation are from the Italian Seismological Instrumental and Parametric Database^68^, time span 2005–2016, colour-coded in map view based on depth. Grey dots are earthquakes filtered out for depth <15 km (upper crust events).

To reconstruct the deeper part of the CSI up to 300 km depth, we extracted a subset of earthquakes with reliable hypocentre locations from the seismicity of the last ten years and analysed them along NW-SE sections (Fig. 7). Below 300 km depth the slab was not investigated in details because seismicity is too sparse.

The slab is well imaged by the seismicity distribution (Fig. 7) and appears continuous down to 70–100 km depth. In the northern sector (Fig. 7, sections 1–9) between 100 km and 150–200 km depth the seismicity is sparse and the position, or even the presence, of the slab cannot be assessed. In this sector the seismicity at depth greater than 200 km is shifted horizontally toward SE with respect to the shallow part of the slab. The gap of seismicity becomes narrower toward the south-west end, and no horizontal shift is observed (Fig. 7 sections 18–20). In the central part of the slab (Fig. 7, sections 10–17), a spatial cluster of seismicity below 70 km depth could indicate an incipient tear along a NNE-SSW, 40° south-east dipping plane with an increasing offset from south to north. Below 150 km depth, the slab may thus be partially disconnected from the rest of the slab above.

### Integrated slab model (5–300 km depth)

**Figure 8:**
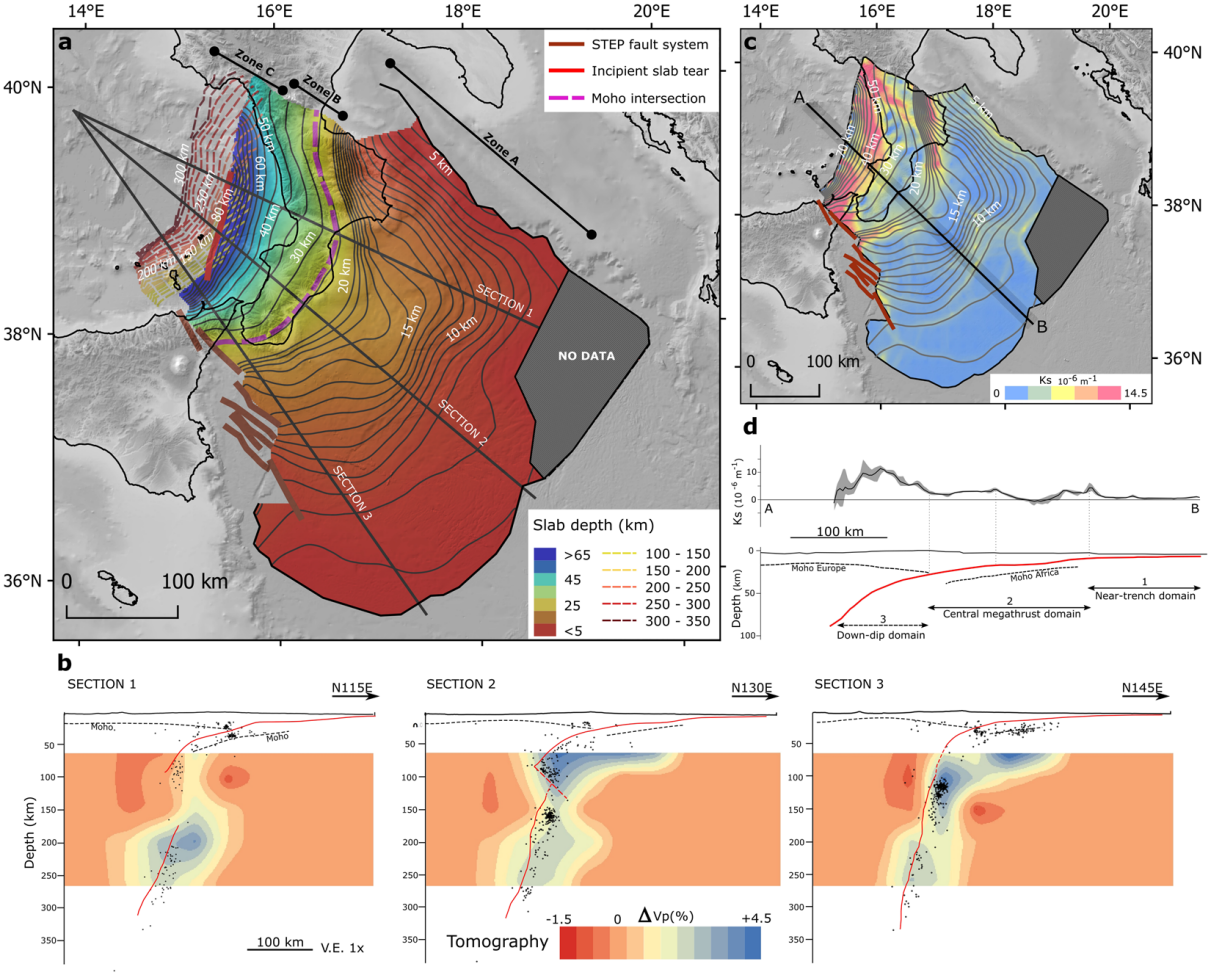
Integrated slab model. Panel a – Gradient-colour map and depth contours of the integrated slab model (smoothing distance = 50 km). Depth contour intervals is 1 km between 5–20 km depth, 5 km between 20–100 km depth, and 10 km from 100 km depth downward. Zone A is the area covered by seismic reflection profiles (Fig. 6), Zone B is the transition zone obtained through interpolation, and Zone C is the subduction interface interpreted from the distribution of instrumental seismicity. The Moho depth^73^ model is from GeoIT3D^74^. Panel b - Sections across the slab. Red line is the position of the subduction interface shown in Panel a. Red dashed line in Section 2 is the incipient slab tear. Black dots are the earthquakes collected in 10-km-wide buffers around the section traces. Dashed black line is the position of the Moho from Scrocca, *et al*.^73^. Tomographic data are from Neri, *et al*.^50^. Panel c - Map of the interface curvature Ks = d*Θ*/d*s*, where *Θ* is the dip angle and *s* is the tangent to the interface^2^. The depth contours from Fig. 8a are shown for reference. Panel d - Swath profile A-B (width = 20 km) of the curvature and a sketch of a possible zonation. Topo-bathymetric relief (Panel a) and profile (Panel c) are obtained from SRTM30_PLUS^72^. Coastlines are from the European Environmental Agency (http://www.eea.europa.eu/).

The slab model presented here (Fig. 8a) results from merging two separate products: a shallower section of the CSI (Zone A in Fig. 8a) and a deeper section (Zone C in Fig. 8a), derived from the analysis of the spatial distribution of earthquakes, illustrated in details in the two previous paragraphs. The interpolation for merging the two sections filled the gap between them (Zone B in Fig. 8a). Notice that the resampling of the shallower section at a smoothing distance of 50 km was necessary to match the lower spatial resolution of the deeper section. Parts of the features described for the shallow region are thus less evident in the integrated slab model because of the smoothing (compare Fig. 6a with Zone A in Fig. 8a).

The integrated slab model (Fig. 8a) shows that in the interval between 20 and 100 km depth the slab is characterized by a rapidly increasing dip with increasing depth. Below, the slab becomes very steep and almost vertical in the south-eastern sector (Fig. 8a). The deeper region in the northern part is disconnected and is horizontally shifted by about 15–20 km to the southeast and rotated clockwise by about 20° with respect to the shallower slab. This feature appears in map view as a partial overlap of the depth contours (Fig. 8a). In our interpretation, this rotation is partially accommodated, in the central part of the slab, by and incipient tear striking NNE-SSW.

The integrated slab model is made available an XYZ spreadsheet for the 0–100 km depth interval and as a shapefile format for contour lines of the 0–300 km depth interval (Supplementary file CSI_ISM.XLS and CSI_ISM_CNT.XLS, respectively).

## Discussion

This work presents a high-quality 3D reconstruction of the CSI (Figs 6 and 8) using an unprecedented dataset of seismic reflection profiles (Fig. 1, and Table 1) for the shallow part and seismicity for the deeper part (Fig. 7).

The seismic reflection dataset allowed us to thoroughly constrain the interpretation in three dimensions taking advantage of the relationships between and among the seismic profiles and to gain a coherent image of the shallow CSI as a whole. However, the absence of well logs in the study area represents the major limitation in order to constrain the local stratigraphy and define the velocity model for the time-depth conversion. The Oceanic Drillings Program sites 964 and KC01 reach only 40 m below the seafloor in this region (Janus Web Database, http://www-odp.tamu.edu/database/). Hence, the adopted stratigraphic scheme was constrained only by the seismic facies and the geologic interpretation of the main reflectors as proposed in other works (Tables 2 and 3).

Two main competing stratigraphic schemes are currently available for this region. In the first scheme the Moho is identified as a continuous high-amplitude reflector at the base of a well-layered, high-amplitude and low-frequency reflector package interpreted as the IOC, overlying a more transparent seismic facies^28, 30^. In the second and more recent scheme the above mentioned well-layered seismic facies is interpreted as a sedimentary succession, probably Mesozoic pelagic carbonates (U4 in Table 2)^32–34^. The internal structure and velocity characteristics of this well-layered reflector package is not consistent with a homogeneous basaltic oceanic crust, which should be characterized by low-reflective to transparent seismic facies with few or none clear acoustic impedance interfaces.

We adopt the most recent scheme also because the internal velocity model proposed for the pre-stack depth-migrated profiles^34^ and the refraction velocity data below  5,000 m/s^61^ support the interpretation of U4 as made of sedimentary rocks^34^. Adopting the earlier scheme would cause an upward shift of the IOC, thus implying a reduced thickness for the overlying sedimentary succession and a shallower Moho. However, this different interpretation does not change significantly the position of the CSI, which is the main target of our reconstruction, because it is picked in the same position as the CROP M2B interpretation of the earlier model^30^. The implications of these two different models of the sedimentary cover thickness mainly impact the amount of subducted IOC that is needed to explain the observed volume of the CAW, but this evaluation is beyond the scope of our work.

The velocity model in Table 4 is thus based on: the velocity proposed in previous studies^33, 34, 61^ for the Plio-Holocene succession (U1) and the Messinian evaporites (U2); the wide-angle seismic data available from the abyssal plain and the accretionary wedge; and the stacking velocities adopted for the processing of the CROP profiles. The instantaneous velocity model adopted for the accretionary wedge (U6) represents a different strategy for the time-depth conversion with respect to the post-stack depth migration adopted in other studies on single seismic profiles. This strategy is needed to consider the 3D time-depth conversion of the entire CSI and the velocity increase with increasing depth related to the compaction of sediments within the CAW. Using a different velocity model from that adopted by us would produce different results for the average depth of the CSI but the main geometrical features would remain the same. However, if better stratigraphic constraints or alternative velocity models should become available in the future, the depth of the shallow CSI can be easily recalculated all at once from our time-domain model. To explore the epistemic uncertainty related with the velocity model, we depth converted our CSI time-domain model with constant velocity values for U6 of 3,500 m/s taken from de Voogd, *et al*.^61^ and 3,100 m/s taken from Gallais, *et al*.^34^ (Supplementary Table S1). The comparison of our CSI depth-domain model with these two alternative reconstructions yields average vertical differences of ~500 m and ~1,000 m, respectively. In general, the uncertainty of the depth-converted CSI is higher in the inner part of the CAW (dashed contours in Fig. 6A) where the quality of seismic reflection profiles is lower (Figs 3, 4 and 5) and the variability related with the adopted velocity model is higher because of the enhanced thickness of the CAW itself (Supplementary Figure S4).

The reconstructed geometry of the shallow CSI (Fig. 6a) together with that of the main structures within the CAW (Supplementary Figures S2 and S3) show that the subduction is confined between two lateral terminations that are very different from one another, thereby suggesting the presence of an asymmetric subduction process in the Calabrian Arc. In the south-western sector, the CSI termination is characterized by the presence of a STEP fault system. The shallow expression of the STEP fault system was originally recognized in bathymetric and high-resolution shallow seismic data acquired in the area^33, 34, 47^. Polonia, *et al*.^33^ suggested that the fault could have cut through the entire CAW, while Gallais, *et al*.^46^ and Gutscher, *et al*.^47^ proposed it to be the major lithospheric tear controlling the subduction of the Ionian slab. Here we show the lateral continuity of the STEP at depth for over 150 km length, and confirm its importance as a lithospheric structure. Our data also suggest that the activation of the STEP fault system should be related to a Plio-Pleistocene tectonic phase, following the emplacement of the Messinian evaporites (U2) because: 1) the post-Messinian wedge (U2), as seen in the shallow external flat region, reaches farther south than the southernmost tip of the STEP fault system (Figs 6 and 2) the shallow expression of this part of the STEP fault system is marked by the presence of Plio-Pleistocene syn-tectonic basins (U1, Fig. 5c). Conversely, in the north-eastern sector the CSI terminates on a lateral ramp that acts as a gradual transition from subduction in the Calabrian Arc to collision in the Southern Apennines. In the northern part of this ramp, the CSI overrides the Apulia Margin (U8) that here trends WNW-ESE (Fig. 6) and is flexured coherently with the CAW (Fig. 5d). The CSI here is also dissected by NW-SE high-angle transpressive faults related to the deformation of the Apulia Margin^38, 40^. These features together suggest that the clockwise rotation of Calabria is accommodated across a diffuse zone of deformation rather than by a STEP fault.

The shallow CSI in between these two different lateral terminations appears as a continuous structural feature. In the NW-SE direction, the CSI has a flat-ramp-flat configuration. With reference to Fig. 3, the flat in the NW part is a detachment running just above the top of U4, a unit identifiable as a low-frequency and low-amplitude seismic facies not involved in the CAW contraction. Above the detachment, U4 is involved in the CAW (U6) contraction with a sort of duplex geometry (Figs 3 and 6c), as already hypothesized by Minelli and Faccenna^32^ by reinterpreting data from Cernobori, *et al*.^28^. Toward the SE the ramp cuts across U3 and connects with the shallow external flat developed in post-Messinian time^33, 34^. This post-Messinian wedge (U2) overrides U3 and U4 which show different seismic facies. In the south-western part, U3 and U4 are generally undisturbed and continuous, whereas in the north-eastern part they are characterized by slightly folded and less continuous reflectors (Supplementary Figures S2 and S3). The enhanced deformation within U3 and U4 occurs in the area where the frontal edge of the CAW is in contact with the Mediterranean Ridge (Fig. 6), whereas the less deformed part occurs in a region where the CAW frontal edge transitions into the undisturbed cover of the IOC. This different level of deformation corresponds to different geomorphic and shallow structural features that led to a subdivision of the CAW into two distinct lobes (north-eastern lobe and south-western lobe)^33, 36^. The transition between these two lobes of the CAW is interpreted by Polonia,* et al*.^36^ as related to the presence of the south-eastward prolongation of the so-called Ionian Fault, identified as a major dextral strike-slip fault running NW-SE from nearby Reggio Calabria to the CAW front, and acting as a STEP^36^. We did not recognize any evidence of the Ionian Fault in our analysis, although a few seismic profiles (#13, #14, and #15; Supplementary Figure S3) are favourably oriented for its detection and assessment of its possible lateral continuity. If the Ionian Fault were a STEP-like fault, it would have been expected to have a major normal offset and secondary right-lateral strike-slip offset cutting the oceanic crust. This area, however, does not show signs of any significant offset. We thus interpret the differences in the seismic facies of U3 and U4 as being related to augmented CAW internal deformation due to the interaction (collision?) with the Mediterranean Ridge in the north-eastern part of the CAW. The Mediterranean Ridge likely works as an obstacle to the south-westward propagation of the CAW north-eastern lobe thereby affecting both the deformation within the CAW and the seismic facies of the Mesozoic and Tertiary sedimentary covers in agreement with Polonia, *et al*.^33^. Hence, the transition between the two lobes within the CAW seems to be an area of diffuse left-lateral strike slip deformation that accommodates two regions of the CAW characterized by different propagation velocities, as previously hypothesized by Polonia*, et al*.^33^ and lately maintained by Gutscher, *et al*.^48^.

In the deeper part of the slab (below 20 km), besides the already well documented slab window in the north^51^, we highlight the potential presence of an incipient slab tear with progressively increasing offset from south to north. We interpret this tear as the ongoing southward propagation of the slab window, as already hypothesized by Wortel and Spakman^62^. This interpretation is well supported by the 3D tomographic model of Neri, *et al*.^50^ which highlights a high-velocity volume whose geometry matches well with the seismicity distribution (Fig. 8b). The Vp anomalies show a slab window between 100 and ~170 km in the northern region (Fig. 8b, Section 1) and supports our interpretation of the continuity of the slab in the southern region (Fig. 8b, Section 3). The shape of the positive Vp anomaly in the central part (Fig. 8b, Section 2) is also in agreement with our interpretation of an incipient slab tear below 70 km depth. The slab depth contours highlight a change in the dip direction between the upper part of the slab and the partially detached, deeper slab. This divergence of the contours suggests a clockwise rotation of the slab at depth that may be related to the mantle dynamics, such as east-directed mantle flow^16^ or toroidal flow through the slab window^12^, and/or elastic rebound due to slab detachment^62, 63^. These processes are prevalent because the slab roll back, that controlled the subduction dynamics since late Miocene, decelerated in the last million years^11^ due to the continental collision of the upper plate with the Apulia platform (northern sector) and to the ensued slab break off.

As regards the integrated slab model, notice that the use of the seismicity distribution for modelling the deeper CSI implies a lower resolution than that of the shallow part. However, the main limitation in this case is the ambiguous information available from seismicity alone between ~18 to ~50 km depth that was covered in our model by interpolation. Future studies should thus be directed at filling this gap. Below 300 km depth our dataset does not allow us to constrain a three dimensional reconstruction, however the presence of the slab is confirmed by several regional tomography studies^49, 64^.

In conclusion, our CSI model provides a reliable geometric constraint for better understanding the behaviour of this subduction zone. Geometric parameters, such as dip, curvature, or roughness are often related to the seismogenic potential of the subduction zones^2, 65^. Figure 8c shows the curvature derived from our integrated slab model and a sketch of possible zonation alternatives of the CSI (Fig. 8d), considering the Moho of the overriding plate as a proxy for the base of the seismogenic zone and curvature changes as its possible upper limits. The average curvature, following the calculation method and relationship proposed by Bletery, *et al*.^2^ yields an estimated maximum moment magnitude of ∼8, which requires an earthquake rupture dimension, using empirical relationships by Strasser, *et al*.^66^, compatible with the overall size of the entire CSI if fully coupled. However, the definition of the seismogenic potential in the Calabrian subduction zone remains one of the main open questions. The capability of generating large earthquakes has often been evoked but never totally agreed upon. The dearth of shallow seismicity related to the CSI can either suggest that it is enduring interseismic locking or is aseismic. The latter would imply that the convergence between the African and Eurasian plates is spent through other mechanisms rather than interplate earthquakes.

## Method

The CSI model presented here was made by merging two separate realizations: a shallower section (Zone A in Fig. 8a) derived from the interpretation of seismic reflection data (dataset shown in Fig. 1 and Table 1), including time-depth conversion and modelling (Fig. 6), and a deeper section (Zone C in Fig. 8a) derived from the analysis of the spatial distribution of earthquakes (Fig. 7). The interpolation for merging the two sections is also used to fill the gap between them (Zone B in Fig. 8a). A resampling of the shallower section at a smoothing distance of 50 km was needed to match the lower spatial resolution of the deeper section. The next paragraphs illustrate the details of the techniques used.

### Interpretation of seismic reflection data

The interpretation of seismic reflection data was primarily devoted to the picking of reflectors that were good candidates to represent the subduction interface and the horizons bounding the stratigraphic units (Fig. 2, Tables 2, and 3) derived from previous studies in the region; secondarily, it was devoted to identify secondary structures (faults and folds) within the CAW. The seismic facies were calibrated in regions of the seismic lines where the succession is relatively undisturbed (i.e., toward the centre of the Ionian basin and in the Apulia foreland).

The subduction interface was identified by visual inspection of the various seismic facies separated by the interface itself and, in particular, by verifying the slope and continuity of seismic reflectors. To reduce the presence of spurious pickings, the line drawings were cross-checked between and among all seismic reflection profiles. The line-drawing procedure was aided by the analysis of seismic-derived attributes (Supplementary Figure S1) as summarized below.

1) The distribution of the normalized signal amplitude versus frequency was used to set up the frequency filters (Supplementary Figure S1). 2) The frequency filters were applied to enhance the signals associated with the different geological bodies. The high-pass filter provided a better display of the Plio-Holocene thin layers where the high-frequency content dominates (Supplementary Figure S1), whereas the low-pass filter enhanced the signals of deeper layers thereby reducing the noise in the profile (Supplementary Figure S1). 3) The similarity index was used to perform a cross-correlation between traces within search windows in the seismic profiles taking into account both the seismic amplitude and the waveform shape. A similarity value equal to 1 indicates that the traces are identical in waveform and amplitude; conversely, a similarity value equal to 0 indicates that they are completely different. The similarity index was used to better constrain the lateral discontinuities of rock bodies, related to the presence of faults (Supplementary Figure S1). 4) The energy attribute was used to enhance the lateral variations within seismic recordings. The application of an *ad hoc* grey-scale palette enhances the display of the main seismic objects; this technique proved to be especially useful for highlighting the separation between the chaotic accretionary wedge and the underlying less deformed seismic units (Supplementary Figure S1). 5) The instantaneous phase was used to emphasize the spatial continuity of reflectors and to highlight the sequence boundaries within the Plio-Holocene unit (U1 in Table 2) and faults (Supplementary Figure S1).

### Velocity modelling and time-depth conversion

The depth conversion of the subduction interface interpreted in the time domain was performed by building a 3D instantaneous velocity model for the entire study area. The velocity model is composed of four layers (Table 4, Fig. 2). The Messinian evaporitic wedge (L3) is not present everywhere. The velocities of the first three layers were considered constant, whereas the fourth layer (CAW) was modelled considering the instantaneous velocity equation of Marsden^67^ with a fixed initial velocity and increasing velocity with increasing depth.

The velocity parameters were derived from the interval stack velocities available for the CROP seismic profiles which were then compared with the wide-angle velocities available in the area for consistency (Supplementary Table S1). The gradient (k), representing the velocity increase with increasing depth, was calculated in nine points located on several seismic reflection profiles (Fig. 2a) and it was then interpolated using the Vel-IO 3D tool^55^ (the contours of the velocity gradient (k) are shown in Fig. 2a).

To perform the 3D depth conversion, the mapped boundary horizons of the four layers were interpolated in the entire area together with the velocity parameters assigned to each layer; the CSI was depth converted using the corresponding velocity parameters at each point, following the approach proposed in Maesano and D’Ambrogi^55^.

### 3D modelling of interpreted seismic reflection data

The depth converted points were imported in the 3D modelling platform of the software MOVE 2016 (Midland Valley Ltd.) and interpolated using a Delaunay triangulation. A 1,500-meter-spaced grid was used for resampling the interpolated surface. The resampled surface was then smoothed in order to remove small-scale features superimposed on the large-scale subduction interface geometry and the noise (anomalous spikes, artefacts) created by spurious data. The optimal smoothing distance was found by firstly taking into account the average spacing distance of the seismic lines (e.g., the average spacing is 50 km for the CA99 survey and smaller for GT08 and MEM07; Fig. 1). Secondly, by performing successive smoothing trials with progressively increasing smoothing distance from 5 to 120 km, at 5 km steps. The average residuals between the originally picked points and the smoothed surfaces, and the average residuals between surfaces obtained with different smoothing distances were then considered. The optimal values were between 10 and 25 km.

### Analysis of the seismicity spatial distribution

For this analysis we used a selection of seismic events taken from ISIDe Working Group^68^. The selection was made according to the following criteria: 1) date of the event more recent than 2005, i.e. since the date that the number of stations has significantly increased in the Italian territory (>300), thereby ensuring that the selected events are presumably those with more accurate locations; hypocentre depth greater than 15 km to exclude earthquakes located in the upper plate; inter-event Euclidean distance shorter than 7.3 km, corresponding to the 90th percentile of the shortest inter-event distance distribution, to exclude sparse seismicity. The application of these criteria led to a selection of 5,710 events with 0.3 ≤ ML ≤ 5.8.

The selected earthquake hypocentres were then normally projected onto three sets of 99 sections, with NW-SE, E-W, and NE-SW orientation and a spacing of 10 km between them, and sampling the events falling within a buffer of 5 km around each section (a sample of 20 NW-SE sections is presented in Fig. 7). The geometry of the slab interface was obtained by fitting the selected seismicity with a 3rd-order polynomial. The fitted curve was translated by few kilometres to ensure that it formed an envelope containing the majority of the events, assumed to represent the intraslab seismicity, and taking care of peculiar features, such as the seismicity gaps, observed in the distribution in each section. The interpreted traces of the subduction interface, which here has a lower resolution than the shallower part, were then interpolated with a grid spacing of 15 km between 20 and 100 km depth and of 20 km for the deeper part ( > 100 km).

## Electronic supplementary material

Supplementary Information

Dataset 1

Dataset 2

Dataset 3

Dataset 4
