# Supplementary material for: The Calabrian Arc: three-dimensional modelling of the subduction interface
Source: Sci Rep. 2017 Aug 21;7:8887. doi: 10.1038/s41598-017-09074-8 (PMC5567100; doi:10.1038/s41598-017-09074-8)
Supplement: Supplementary file 1 — Supplementary Information [file 41598_2017_9074_MOESM1_ESM.pdf]

SUPPLEMENTARY INFORMATION

The Calabrian Arc: three-dimensional modelling of the subduction interface

Francesco Emanuele Maesano*, Mara Monica Tiberti, and Roberto Basili

Istituto Nazionale di Geofisica e Vulcanologia, Via di Vigna Murata 605, 00143 Rome, Italy.

The supplementary information include this file and the file SupplementaryData.zip.

SupplementaryData.zip includes:

1. The time-domain model of the CSI as an XYZ spreadsheet (CSI_TDM.XLS).
2. The depth-domain model of the CSI as an XYZ spreadsheet (CSI_DDM.XLS).
3. The integrated slab model of the CSI for the 0-100 km depth interval as XYZ spreadsheet (CSI_ISM.XLS), and contour lines vertexes for the 0-300 km depth interval as XYZ spreadsheet (CSI_ISM_CNT.XLS).

In the following, the figures in order of appearance.

Figure S1 | Seismic-derived attributes.

Seismic attributes extracted from the SEG-Y data and used in this study for the interpretation of seismic reflection data. Panel a - Frequency content of the seismic line shown in Panels b-d (profile #7 in Supplementary Figure S2). Panel b – High-pass filter with 20 Hz threshold. Panel c – Low-pass filter with 20 Hz threshold. Panel d – Similarity attribute, colour scale from black (similarity = 0) to white (similarity = 1). Panel e – Energy attribute. Panel f – Instantaneous phase attribute.

Figure made using OpendTect 6.0 (GPL v3 license, https://www.opendtect.org/) and Inkscape 0.91 (https://inkscape.org/it/).

Figure S2 | Line drawing profiles and CSI time-domain model.

Panel a - Line drawing of profiles #1-8. Horizon colour codes as in Figure 2. Yellow lines: unconformities within the Plio-Holocene basins (U1). Description of units (U1-9) and bounding horizons (H1-7) are reported in Table 2 and Table 3, respectively. Legend for the seismic line location map as in Figure 1. Panel b – Dataset used in this study. Panel c – Interpolation of the CSI in time domain (two-way time).

Raw seismic data provided by Spectrum Geo (http://www.spectrumgeo.com/). Figure made using Move 2016.1 (http://www.mve.com/software/move), Kingdom Suite 2016 (https://www.ihs.com/products/kingdom-seismic-geological-interpretation-software.html), OpendTect 6.0 (GPL v3 license, https://www.opendtect.org/), QGIS 2.16 (QGIS Development Team, 2016. QGIS Geographic Information System. Open Source Geospatial Foundation Project. http://www.qgis.org/), and Inkscape 0.91 (https://inkscape.org/it/).

Figure S3 | Line drawing profiles.

Line drawing of profiles #11 and #13-15. All symbols and profile locations as in Figure S2.

Raw seismic data provided by Spectrum Geo (http://www.spectrumgeo.com/). Figure made using Move 2016.1 (http://www.mve.com/software/move), Kingdom Suite 2016 (https://www.ihs.com/products/kingdom-seismic-geological-interpretation-software.html), OpendTect 6.0 (GPL v3 license, https://www.opendtect.org/), QGIS 2.16 (QGIS Development Team, 2016. QGIS Geographic Information System. Open Source Geospatial Foundation Project. http://www.qgis.org/), and Inkscape 0.91 (https://inkscape.org/it/).

Table S1 | Velocity models

Alternative velocity models for the study area. This work adopt an instantaneous velocity model for the accretionary wedge (see Maesano and D'Ambrogi [1] for details on the instantaneous velocity model approach). The velocity model of de Voogd, et al. [2] is derived from wide angle seismic data. The velocity model of Gallais, et al. [3] is derived from the collection of literature data and analysis of original data. Valenti [4] provides a collection of literature data on the velocity model of the Messinian evaporites.

Figure S4 | Sensitivity analysis of the depth conversion

Analysis of the effects of using different velocity models to depth convert the CSI time-domain model (see Figure S2c). The CSI depth-converted using the velocity model proposed in this work (MA17) is compared with the CSI depth-converted using: 1) the velocity model from de Voogd, et al. [2] (DV92); and 2) the velocity model from Gallais, et al. [3] (GA12). See Table S1 for details of these velocity models. The vertical difference is calculated as Δz=MA17-DV92 (Panels a and b), and Δz=MA17-GA12 (Panels c and d). Positive Δz values indicate where MA17 is deeper. Panel e – Histogram and cumulative distribution of the normalized frequency of Δz for the two model comparisons. Panel f – Histogram of the normalized frequency of Δz relative to the CSI depth presented in this study. The relative vertical difference is independent of the depth of the CSI.

Figure made using QGIS 2.16 (QGIS Development Team, 2016. QGIS Geographic Information System. Open Source Geospatial Foundation Project. http://www.qgis.org/), ESRI ArcMap 10.3.1 (http://www.esri.com/software/arcgis/arcgis-for-desktop) and Inkscape 0.91 (https://inkscape.org/it/).

References

1 Maesano, F. E. & D'Ambrogi, C. Vel-IO 3D: A tool for 3D velocity model construction, optimization and time-depth conversion in 3D geological modeling workflow. *Computers & Geosciences* **99**, 171-182, doi:10.1016/j.cageo.2016.11.013 (2017).

2 de Voogd, B. *et al.* Two-ship deep seismic soundings in the basins of the Eastern Mediterranean Sea (Pasiphae cruise). *Geophysical Journal International* **109**, 536-552 (1992).

3 Gallais, F., Gutscher, M.-A., Klaeschen, D. & Graindorge, D. Two-stage growth of the Calabrian accretionary wedge in the Ionian Sea (Central Mediterranean): Constraints from depth-migrated multichannel seismic data. *Marine Geology* **326-328**, 28-45, doi:10.1016/j.margeo.2012.08.006 (2012).

4 Valenti, V. Shallow structures at the outer Calabrian accretionary wedge (NW Ionian Sea): new insights from recently migrated reflection data. *Terra Nova* **22**, 453-462, doi:10.1111/j.1365-3121.2010.00964.x (2010).

Figure S1

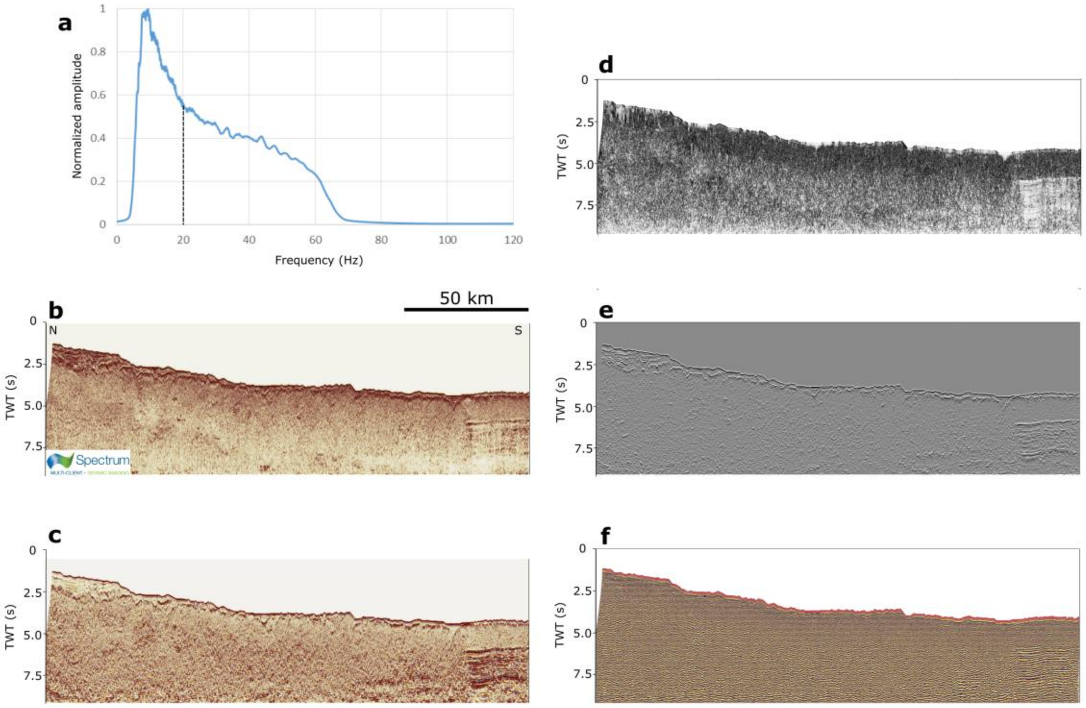

a
Normalized amplitude
Frequency (Hz)
0
0.2
0.4
0.6
0.8
1
0
20
40
60
80
100
120
b
N
S
50 km
TWT (s)
0
2.5
5.0
7.5
Spectrum
c
TWT (s)
0
2.5
5.0
7.5
d
TWT (s)
0
2.5
5.0
7.5
e
TWT (s)
0
2.5
5.0
7.5
f
TWT (s)
0
2.5
5.0
7.5

Figure S2

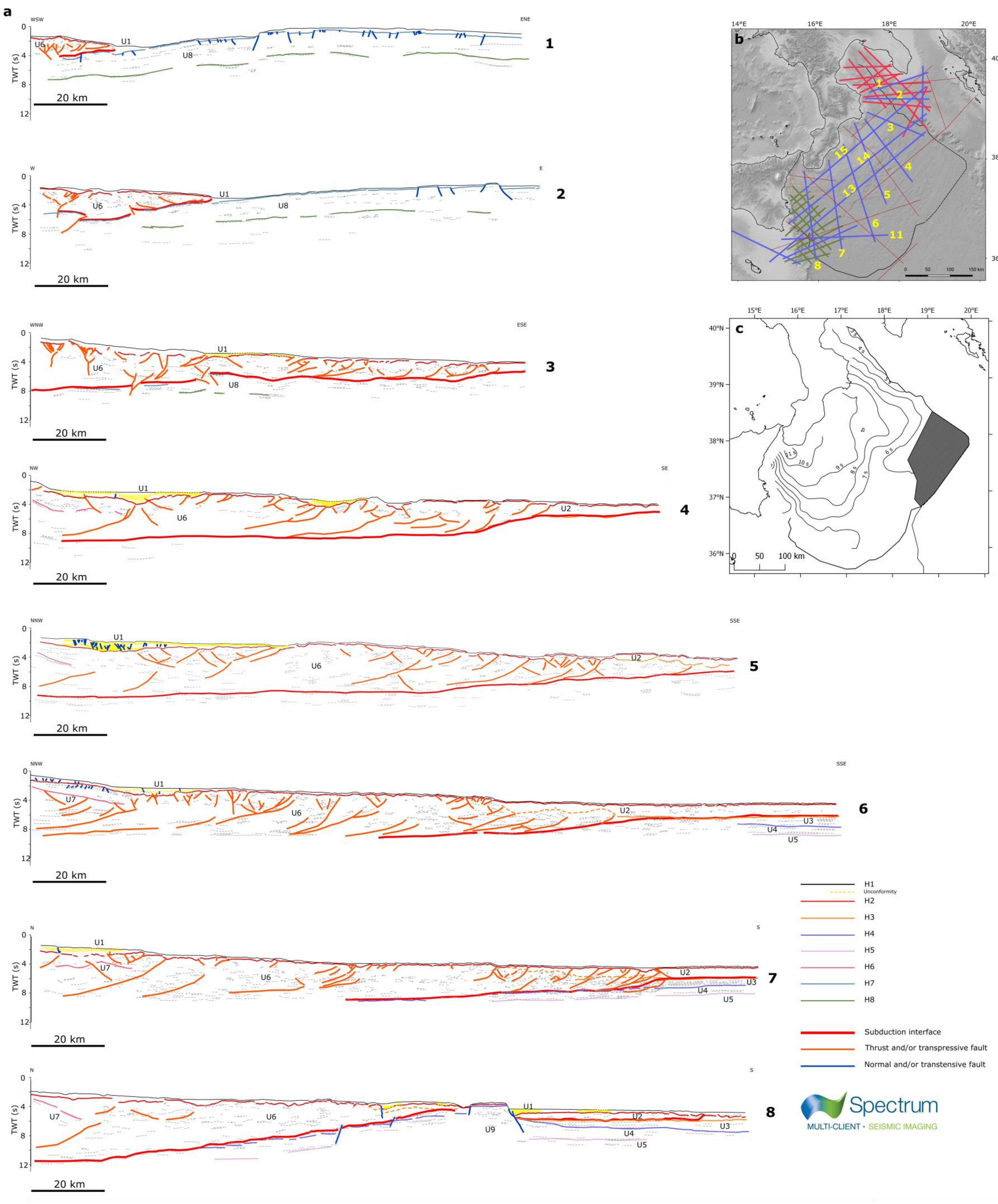
a
b
c
TWT (s)
20 km
WSW
ENE
W
E
WNW
ESE
NW
SE
NNW
SSE
N
S
U1
U2
U3
U4
U5
U6
U7
U8
U9
1
2
3
4
5
6
7
8
14°E
16°E
18°E
20°E
40°N
38°N
36°N
15°E
17°E
19°E
39°N
37°N
0 50 100 150 km
0 50 100 km
H1
Unconformity
H2
H3
H4
H5
H6
H7
H8
Subduction interface
Thrust and/or transpressive fault
Normal and/or transtensive fault
Spectrum
MULTI-CLIENT · SEISMIC IMAGING

Figure S3

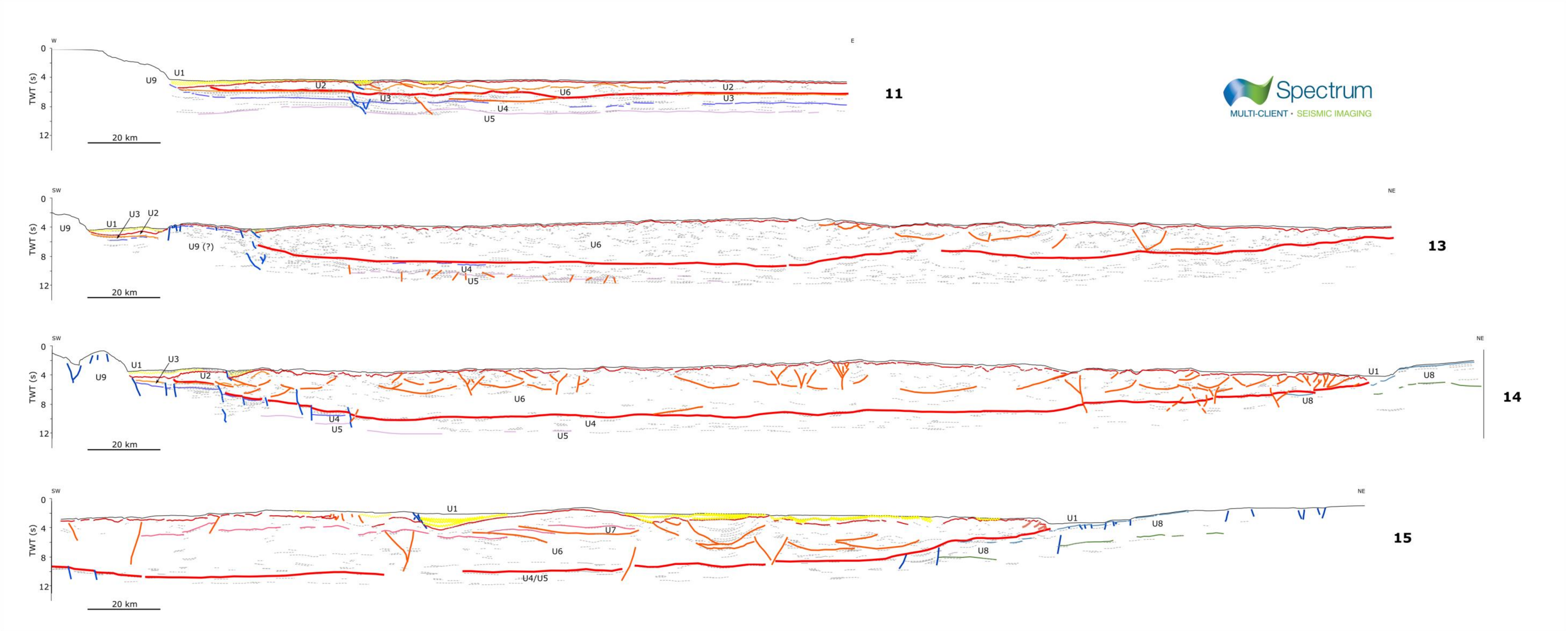

W
E
TWT (s)
0
4
8
12
U1
U9
U2
U3
U4
U5
U6
20 km
11
Spectrum
MULTI-CLIENT • SEISMIC IMAGING
SW
NE
U9 (?)
13
14
U8
15
U7
U4/U5

Table S1

Table S1 | Velocity models							
			**This work**		**de Voogd et al. (1992)**[2]	**Gallais et al. (2012)**[3]	**Valenti (2010)**[4] **and references therein**
**Layer**	**Layer name**	**Unit**	**V (m/s)**	**K (1/s)**	**V (m/s)**	**V (m/s)**	**V (m/s)**
L1	Water	n.a.	1500	0	1500	1500	
L2	Plio-Pleistocene unit	U1	1900	0	1900	1800-2000	
L3	Messinian wedge	U2	4000	0	4200	4200-4500	3000-4500
L4	Pre-Messinian wedge and units	U6	2500	0.15-0.50	3000-3800	3000-3200	

Figure S4

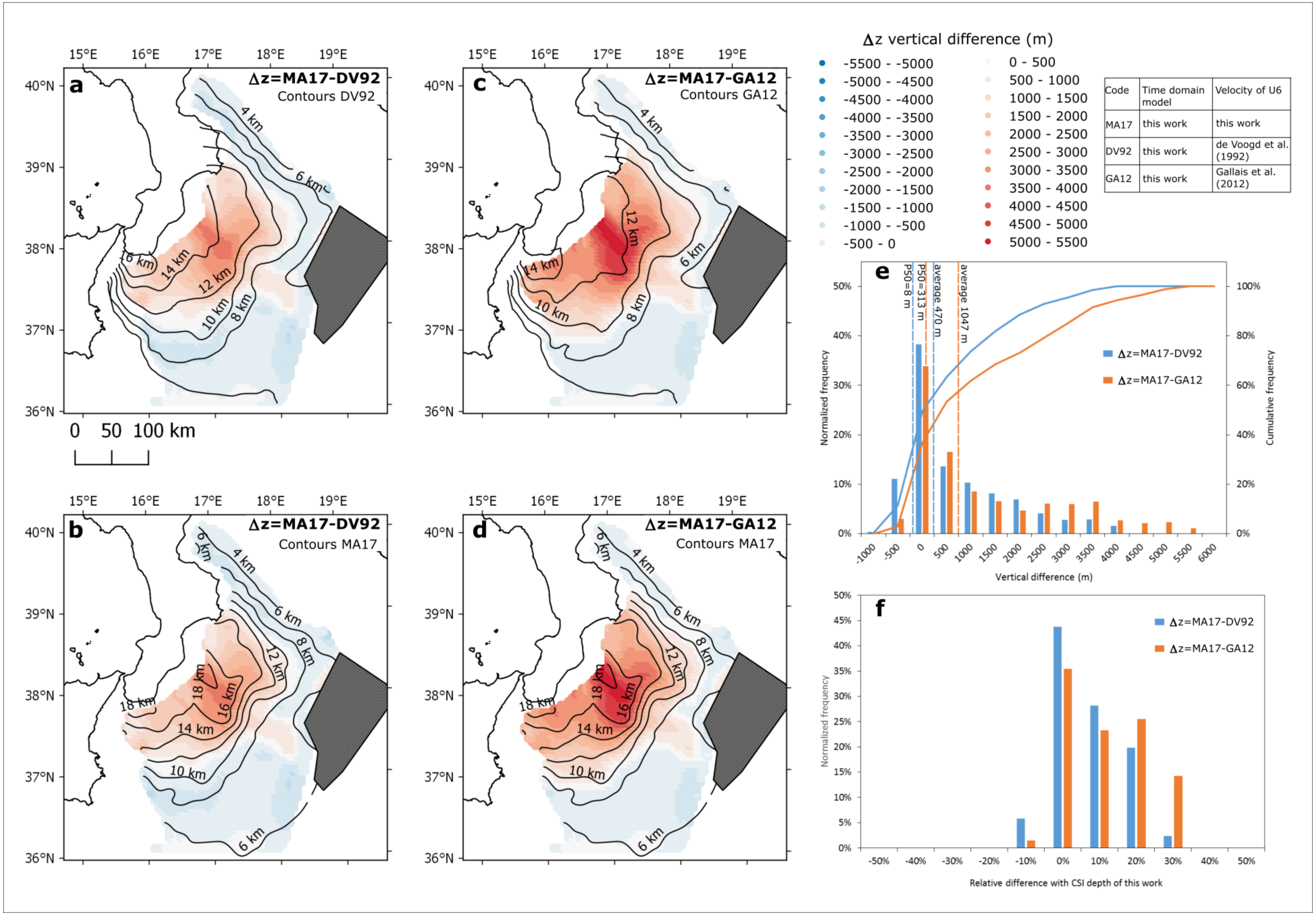

Code	Time domain model	Velocity of U6
MA17	this work	this work
DV92	this work	de Voogd et al. (1992)
GA12	this work	Gallais et al. (2012)
